# The role of the notochord in amniote vertebral column segmentation

**DOI:** 10.1016/j.ydbio.2018.04.005

**Published:** 2018-07-01

**Authors:** Lizzy Ward, Angel S.W. Pang, Susan E. Evans, Claudio D. Stern

**Affiliations:** Department of Cell and Developmental Biology, University College London, Gower Street, London WC1E 6BT, U.K.

## Abstract

The vertebral column is segmented, comprising an alternating series of vertebrae and intervertebral discs along the head-tail axis. The vertebrae and outer portion (annulus fibrosus) of the disc are derived from the sclerotome part of the somites, whereas the inner nucleus pulposus of the disc is derived from the notochord. Here we investigate the role of the notochord in vertebral patterning through a series of microsurgical experiments in chick embryos. Ablation of the notochord causes loss of segmentation of vertebral bodies and discs. However, the notochord cannot segment in the absence of the surrounding sclerotome. To test whether the notochord dictates sclerotome segmentation, we grafted an ectopic notochord. We find that the intrinsic segmentation of the sclerotome is dominant over any segmental information the notochord may possess, and no evidence that the chick notochord is intrinsically segmented. We propose that the segmental pattern of vertebral bodies and discs in chick is dictated by the sclerotome, which first signals to the notochord to ensure that the nucleus pulposus develops in register with the somite-derived annulus fibrosus. Later, the notochord is required for maintenance of sclerotome segmentation as the mature vertebral bodies and intervertebral discs form. These results highlight differences in vertebral development between amniotes and teleosts including zebrafish, where the notochord dictates the segmental pattern. The relative importance of the sclerotome and notochord in vertebral patterning has changed significantly during evolution.

## Highlights

• The chick notochord has no intrinsic segmentation.
• Neither notochord or sclerotome can segment normally without the other.
• The notochord attracts sclerotome cells to the midline.
• Vertebral segmentation involves reciprocal signals between notochord and sclerotome.

## Graphical abstract

**Figure:**
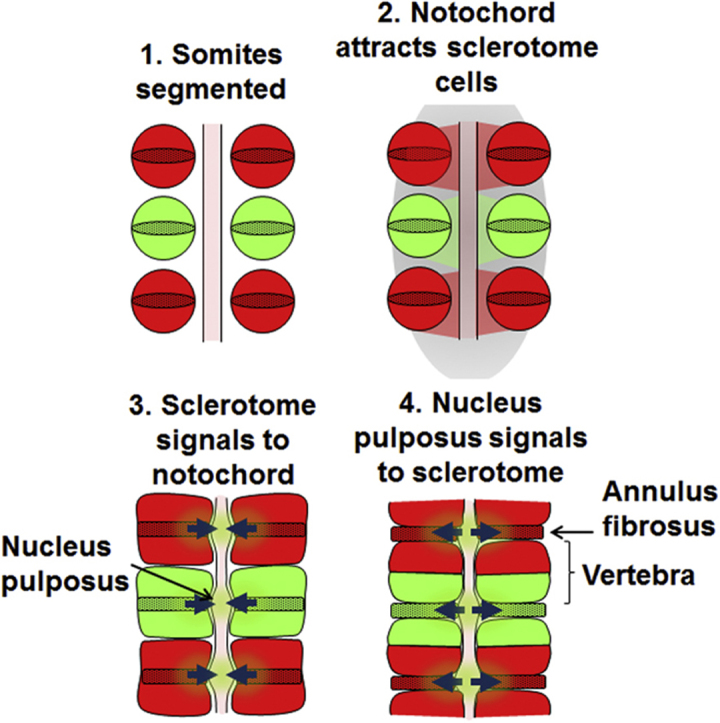

## 1 Introduction

All vertebrates have a segmented body plan, in which the major tissues of the body are divided into a series of repetitive units along the head-tail (or rostro-caudal, R-C) axis. The vertebral column, the defining feature of the clade, is one such segmented tissue. It is comprised of a series of vertebrae, each separated by an intervertebral disc (IVD). The number of segments varies considerably between species. Frogs, for example, can have as few as nine vertebrae, whilst some snakes possess over 300 (Burke et al., 1995, Richardson et al., 1998). The relative size and morphology of each vertebra is also variable both between species and from segment to segment in the same individual. To understand how this remarkable diversity in spine morphology is achieved, we must look to the developmental mechanisms that govern the subdivision of the body into segments.

The vertebral column is derived from two embryonic tissues, the somites and the notochord. The somites are the first visible sign of segmentation in the embryo and form from the pre-somitic mesoderm (PSM) on either side of the midline. As well as giving rise to the vertebral column, the somites contain the precursors of the skeletal muscles and dermis of the trunk, as well as some connective tissue and blood vessel endothelium (Christ and Ordahl, 1995, Christ and Scaal, 2008). Upon formation, each somite consists of an epithelial sphere of cells surrounding a central lumen and is patterned into rostral and caudal halves, which differ in cell density and molecular properties (Stern et al., 1986, Norris et al., 1989, Krull et al., 1997). This R-C patterning is crucial for somite segmentation: the cells from adjacent half-sclerotomes are non-miscible and when half-sclerotomes of the same R-C identity are grafted in close proximity, the cells mix, disrupting segmentation (Stern and Keynes, 1987). The spinal nerves and streams of neural crest cells from the neural tube are only permitted to migrate through the rostral half of each somite (Keynes and Stern, 1984, Rickmann et al., 1985, Bronner-Fraser, 1986, Bronner-Fraser et al., 1991), thereby conferring a segmental pattern upon the peripheral nervous system. As a result, somites lay the foundation on which the final segmental pattern of the animal is built.

In contrast to the somites, the amniote notochord has no visible sign of segmentation upon its formation, consisting of a rod of mesoderm running along the midline of the embryo. Notochord cells are vacuolated, creating an outward force of osmotic pressure that is resisted by a sheath of extracellular matrix (ECM), making it both strong and flexible (Adams et al., 1990, Stemple, 2005). In non-vertebrate chordates the notochord persists into adulthood as the primary axial structure of the animal (Gee, 1996, Delsuc et al., 2006). In vertebrates, however, this structural role is taken over by the vertebral column. Shortly after formation, each somite differentiates into two populations of cells: the sclerotome, which gives rise to the vertebral column and ribs, and the dermomyotome, which gives rise to the trunk skeletal muscles and dermis. Sclerotome cells then undergo epithelial to mesenchymal transition (EMT) and migrate medially to surround the notochord and neural tube, condensing into a segmented pattern that will generate the vertebrae and contribute to the IVD. In most amniotes, the vertebral bodies completely replace the notochord. In these animals, the notochord persists only as the central portion (nucleus pulposus, NP) of the IVD (Walmsley, 1953, Choi et al., 2008, McCann et al., 2012). The outer portion of the IVD, the annulus fibrosus (AF), is derived from the sclerotome. Therefore, the notochord eventually segments and adopts a periodicity that aligns with that of the sclerotome. How is this alignment achieved?

Vertebral segmental patterning can be viewed as a two-step process: (1) Establishment of an initial segmental pattern (somitogenesis) and (2) translation of this pattern to the final arrangement of vertebrae and IVDs. Somitogenesis itself is thought to be regulated by a cell-autonomous process. Pairs of somites sequentially “bud off” from the anterior PSM in rostral-to-caudal direction along the axis in a highly regulated and rhythmic fashion. The number, size and/or R-C subdivision of the somites that form are regulated by a complex molecular oscillator comprised of a set of ‘segmentation clock’ genes (mainly components and targets of the Notch, Wnt and FGF pathways) that exhibit oscillatory expression within cells of the PSM (Palmeirim et al., 1997, Schroter and Oates, 2010, Pourquie, 2011, Oates et al., 2012, Harima et al., 2013, Dias et al., 2014).

This initial pattern must then be translated into that of the vertebral column. However, there is not a 1:1 relationship between somite and vertebral segments. Cell tracing studies have shown that each vertebra is derived from cells from the caudal half of one sclerotome and the rostral half-sclerotome of the somite immediately caudal to it (Bagnall et al., 1988, Huang et al., 1996, Huang et al., 2000, Aoyama and Asamoto, 2000, Ward et al., 2017), a process known as “resegmentation” (Remak, 1855). Consequently, vertebral segmentation is offset with respect to the somites by half a segment and the boundary between two vertebrae (the position of the IVD) sits somewhere in the middle of the original somitic segment. This process establishes the periodicity of the vertebral bodies and IVDs; however it is still not clear how the position of vertebral boundaries (and therefore the spacing between them) is determined.

Given that the rostral and caudal sclerotome halves contribute to different vertebrae, it would be reasonable to suggest that R-C patterning of the somite is important for resegmentation. However, analysis of Mesp1/2 and Ripply1/2 mutants in mouse suggest more complexity. These genes act as a molecular switch to establish and maintain R-C compartment boundaries in the somite (Saga et al., 1997, Morimoto et al., 2007, Takahashi et al., 2010). When either of these genes is knocked out, R-C polarity of the somite is lost. In these knockout mice, periodicity of the IVDs and vertebral bodies is disrupted but not completely abolished, with segmentation defects variable in their severity along the axis (Takahashi et al., 2013). If R-C patterning is completely abolished in these mutants, this could indicate that some aspects of mouse vertebral body and IVD segmentation do not rely solely on R-C patterning of the somites. Could the notochord play a role in segmental patterning (Stern, 1990)? Consistent with this, it has been reported that notochord ablation in chick has no effect on the pattern of neural arches, but the vertebral bodies and IVDs are replaced by an unsegmented strip of cartilage (Watterson et al., 1954, Strudel, 1955). This suggests that the notochord may have an influence on segmental patterning of the vertebral column in chick.

Here we study the role of the notochord on segmental patterning further. Through a series of surgical manipulations, we test the relative influence of the notochord and somites on vertebral segmentation. We show that although the notochord can alter the segmental periodicity of the sclerotome, the sclerotome is the dominant factor influencing segmentation in the early vertebral column. Through reciprocal ablation experiments, we show that the sclerotome cannot form segmented vertebral bodies and IVDs in the absence of a notochord, nor can the notochord segment in the absence of adjacent sclerotome. We propose that the alternation of vertebral bodies and IVDs is regulated by sequential events involving cross-talk between the sclerotome and the notochord, to ensure that both tissues segment in register.

## 2 Results

### 2.1 Vertebral body segmentation is lost when the notochord is ablated

**Fig. 1:**
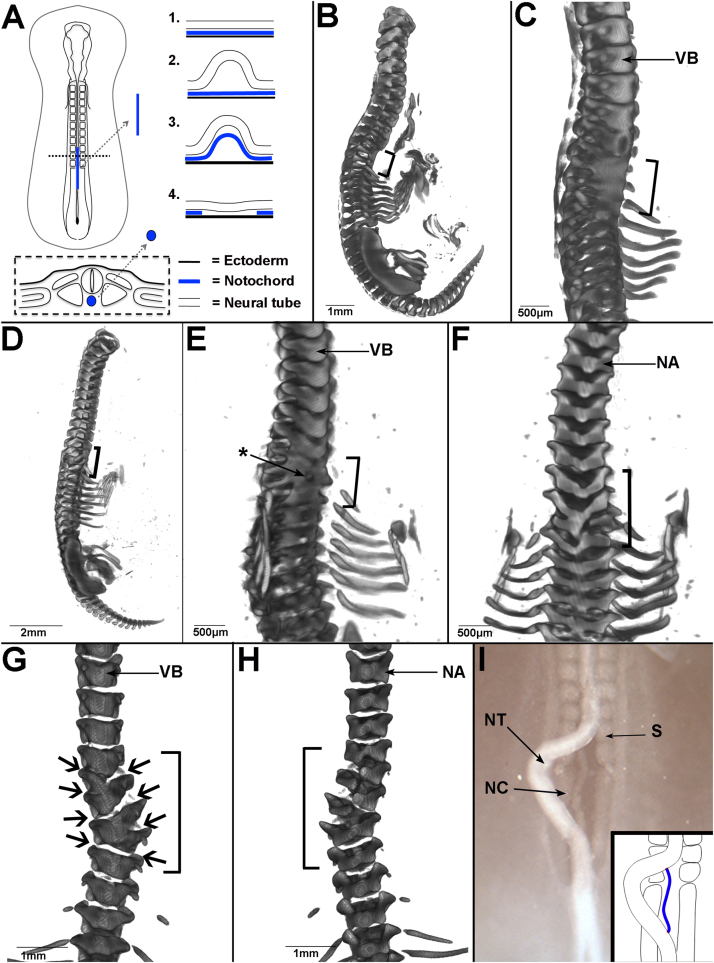
Vertebral body segmentation is lost when the notochord is ablated. A. Schematic showing the notochord ablation procedure. (Above left = dorsal view, below = transverse section; Above right=sagittal view of steps 1–4 of the ablation procedure). B–F. OPT reconstruction of two HH30–32 embryos, six days after notochord ablation and skeletal preparation (black brackets = ablated region). B. First example, whole embryo (head removed). C. First example, ventro-lateral view of vertebral bodies of embryo in B. Zoomed on ablated region. D. Second example, whole embryo (head removed). E. Second example, ventro-lateral view of vertebral bodies of embryo in D. Zoomed on ablated region. (Star = hole/foramen). F. Second example, dorsal view of neural arches of embryo in D. Neural arches show abnormal morphology and disrupted segmentation. G–H**.** OPT reconstruction of HH30–32 embryo skeletal preparations, six days after a sham notochord ablation (black brackets = operated region; black arrows= position of intervertebral discs). G. Sham ablated embryo, ventral view, zoom on operated region. Vertebral bodies do not fuse, but IVDs are misaligned on either side of the midline. H. Dorsal view of embryo in G, showing misalignment of neural arches in the operated region. (NA= neural arch, VB= vertebral body). I. Bright-field image of embryo immediately after sham ablation operation, showing that the notochord does not sit parallel to the midline after being raised from the endoderm. Inset image shows illustration of this, with notochord in blue (NT=neural tube, S=somite, NC= notochord).

To test whether the notochord is required for vertebral body segmentation, the notochord was ablated prior to sclerotome formation and the morphology of the vertebral column analysed six days later. A portion of notochord 4–6 somites in length was excised from the cervical/ thoracic region of HH 10–12 chick embryos (Fig. 1A). Embryos were harvested at HH 32–33, skeletal preparations made and analysed by optical projection tomography (OPT). In 7/7 embryos the vertebral bodies were replaced by a fused strip of cartilage in the ablated region, indicating loss of segmentation (Fig. 1B-E). Segmentation of the neural arches was maintained in all embryos; 3/7 embryos showed normal neural arch morphology throughout the vertebral column (Fig. 1B). The remaining 4/7 showed some degree of disruption to neural arch morphology including fusions and/or a misalignment of the left and right arches (Fig. 1D, F).

Control (sham) notochord ablation experiments were also carried out. Skeletal preparations showed that vertebral body segmentation was maintained in the operated region (3/3 embryos). In 1/3 of these, vertebral morphology was completely normal. In the other 2/3, the vertebral bodies showed misalignment between right and left halves and lateral bending of the spine (scoliosis) (Fig. 1G, arrows). Although misaligned, there were clear spaces in Alcian blue staining, corresponding to the position of the IVDs (Fig. 1G; black arrows). In these embryos, neural arch morphology was also abnormal (Fig. 1H), suggesting that the misalignments and fusions of neural arches in experimental embryos was the result of disruption to the neural tube (Strudel, 1955). A possible explanation for the misalignment is that when the notochord and neural tube are placed back into position, they do not lie exactly parallel to the midline (Fig. 1I). This may result in a shift of the sclerotome on either side, causing a misalignment of the right and left half of the vertebrae.

Together, these results support the idea that the notochord is required for normal segmentation of the vertebral bodies and IVDs but not the neural arches, as reported (Watterson et al., 1954, Strudel, 1955).

### 2.2 An ectopic notochord can alter the periodicity of host sclerotome

**Fig. 2:**
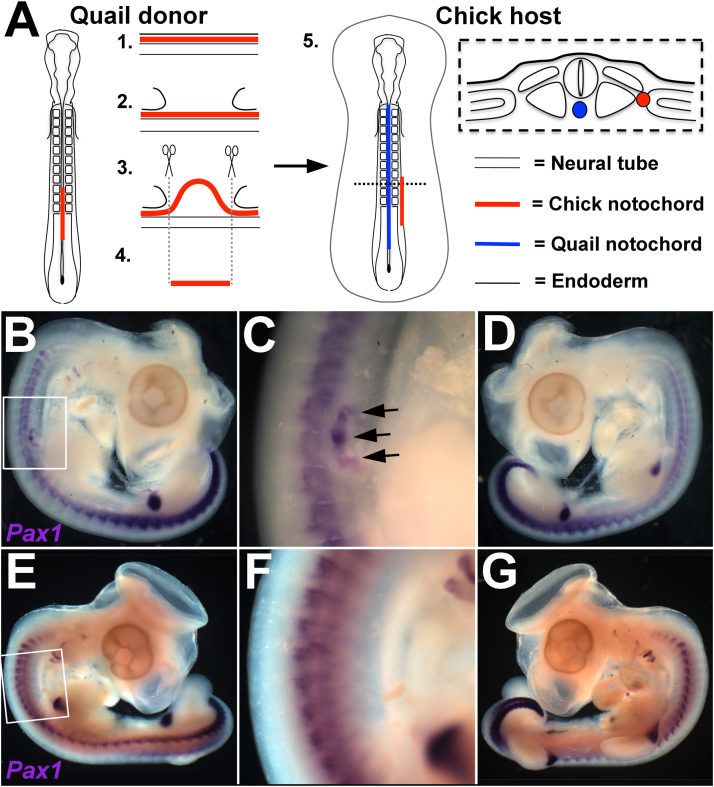
An ectopic notochord graft leads to the formation of ectopic sclerotome lateral to the endogenous somites. A. Schematic showing the notochord graft procedure. (Left = quail donor, centre left = sagittal view of steps 1–4 of notochord graft procedure; centre right = chick host with quail notochord grafted lateral to the host somites (step 5); right = transverse section of chick host after grafting). B–D. WMISH for *Pax1* (a marker of the sclerotome) in a HH stage 25 embryo, three days after a notochord graft. Ectopic *Pax1* expression is seen in the grafted region anterior to the forelimb (B–C; black arrows). No ectopic *Pax1* expression is seen on the contralateral side of the embryo (D). E–G. WMISH for *Pax1* in a HH stage 25 embryo, three days after a ‘sham’ notochord graft. No ectopic expression is seen in the operated region (E-F) or on the contralateral side of the embryo (G).

Next, we tested whether the notochord can influence the segmental pattern of the sclerotome. An ectopic notochord from the posterior cervical region of HH 10–11 quail embryos was grafted lateral to the cervical somites of chick hosts of the same stage (Fig. 2A). Three days later (HH 24–25), whole-mount in situ hybridisation (WMISH) for the sclerotome marker Pax1 showed the presence of ectopic sclerotome in the grafted region just rostral to the forelimb (9/13) (Fig. 2B, C; black arrows). In all but one embryo, there was no change either to the size or to the periodicity of the endogenous sclerotome on the graft side (Fig. 2B, C) or the contralateral side of the embryos (Fig. 2D). In 4/4 sham operated control embryos, Pax1 expression was normal on both sides of the embryo (Fig. 2E–G).

**Fig. 3:**
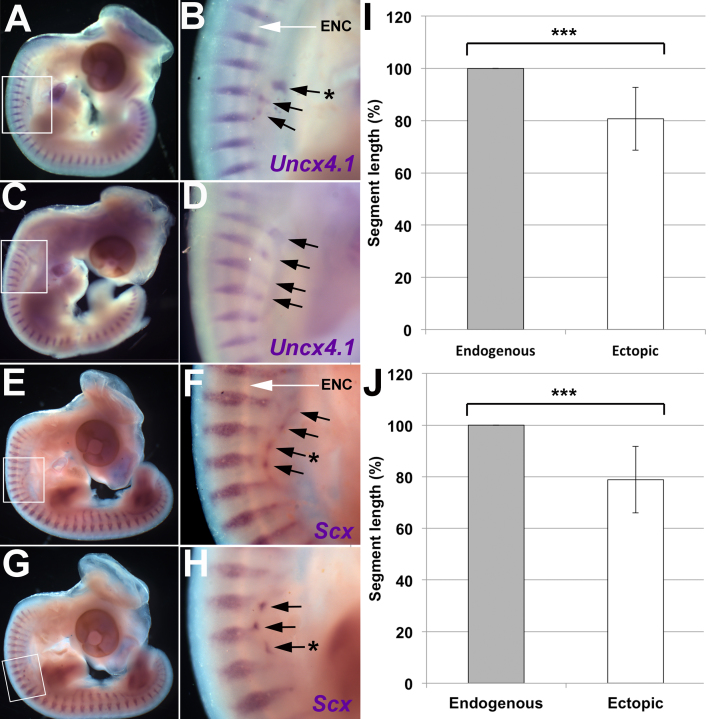
A notochord graft leads to the formation of ectopic sclerotome with a different segmental periodicity to host sclerotome. A–D. WMISH for *Uncx4.1* in HH stage 24–25 embryos three days after a notochord graft. Ectopic *Uncx4.1* expression is seen in the region of the notochord graft. A-B. Example one. C–D. Example two. E-H. WMISH for *Scleraxis* in HH stage 24-25 embryos three days after a notochord graft. Ectopic *Scleraxis* expression is seen in the region of the notochord graft. E-F. Example one. G-H. Example two. I. Comparison of endogenous and ectopic segment length in *Uncx4.*1-stained embryos. Ectopic segments are an average of 19% smaller than the adjacent endogenous segments. A pairwise student T-test shows this difference is statistically significant (p < 0.005, n = 14). J. Comparison of endogenous and ectopic segment length in *Scleraxis*-stained embryos. Ectopic segments are an average of 21% smaller compared to the adjacent endogenous segment. (Black arrows = segments of ectopic expression, Black star = segments of expression that are significantly out of phase with the endogenous expression pattern, ENC= endogenous notochord visible as white stripe extending from A-P along the axis).

To analyse the segmental periodicity of the ectopic sclerotome, notochord-grafted embryos at HH 24–25 were stained by WMISH for Uncx4.1, a marker of the caudal sclerotome (Mansouri et al., 1997, Neidhardt et al., 1997) and Scleraxis (Scx), which marks a sub-compartment of tendon progenitors within the sclerotome (Brent et al., 2003). Ectopic expression of both markers was seen in the grafted region (Uncx4.1: 6/7 embryos; Scx: 4/4 embryos), revealing that segments were closer together in the ectopic sclerotome compared to the adjacent endogenous sclerotome (Uncx4.1: 5/6 embryos; Scx: 4/4 embryos) (Fig. 3A-H; black arrows). Furthermore, several of the ectopic stripes were not aligned with the endogenous segments (Fig. 3B, F, H; black star). The observed difference in sclerotome periodicity was verified by measuring segment length (distance between successive expression domains). In Uncx4.1-stained embryos, ectopic segments were an average of 19 ± 12% (S.D.) shorter than that of the adjacent endogenous sclerotome (Fig. 3I). Similarly, in Scx-stained embryos, ectopic segments were 21 ± 13% shorter than that of the adjacent endogenous sclerotome (Fig. 3J). Pairwise Student's T-tests showed this to be statistically significant (Uncx4.1: t(10) = 5.53, p = 0.000251; Scx: t(13) = 6.21, p = 0.000032).

**Fig. 4:**
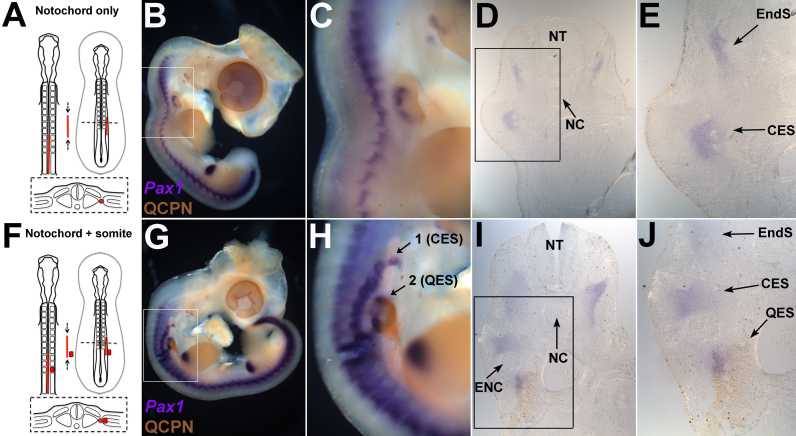
Ectopic sclerotome is derived from the host. A–E. Notochord graft only. A. Schematic of notochord graft procedure. B. WMISH for sclerotome marker *Pax1* (purple) and immunohistochemical stain for the quail-specific marker QCPN (brown) in HH stage 24/25 embryos, three days after a notochord graft. C. Higher magnification of boxed region in B. D. Transverse section of embryo in B shows endogenous and ectopic sclerotome. E. Higher magnification of boxed region in D. Ectopic sclerotome contains no QCPN-positive staining, showing it is derived from the chick host. F–J. Notochord plus somite graft. F. Notochord plus somite graft procedure. G. WMISH for *Pax1* and immunohistochemical stain for QCPN in HH stage 24/25 embryos, three days after a notochord and somite graft. H. Higher magnification of boxed region in G. Two rows of ectopic sclerotome can be seen. I. Transverse section of embryo in G showing endogenous sclerotome and two rows of ectopic sclerotome dorsal and ventral to the ectopic quail notochord. J. Higher magnification of boxed region in I. Ectopic sclerotome below the quail notochord graft contains QCPN-positive cells, showing it is derived from the grafted somite. (NT=neural tube, NC=endogenous notochord, ENC=ectopic quail notochord, CES=chick-derived ectopic sclerotome, QES=quail-derived ectopic sclerotome, EndS=endogenous sclerotome).

These results suggest that the grafted notochord somehow compresses the periodicity of the host sclerotome. However, it is possible that the Pax1-positive cells seen in the grafted region are not derived from the host, but from quail sclerotome cells that had been transferred accidentally with the donor notochord. Since QCPN antibody does not stain notochord nuclei at this stage, some embryos were grafted with both a notochord and a single quail somite (Fig. 4F) to act as positive controls for QCPN staining. In these control embryos with a notochord and somite graft, two populations of ectopic sclerotome were seen (2/3 embryos; Fig. 4G, H; arrows 1 and 2). Transverse sections revealed that a proportion of the Pax1-expressing cells were QCPN-positive, indicating that they were derived from the grafted quail somite (Fig. 4I, J). This confirms that QCPN staining can successfully detect quail-derived sclerotome cells. The remaining Pax1-positive cells were QCPN-negative, and therefore derived from the chick host (Fig. 4I, J; CES=chick ectopic sclerotome, QES=quail ectopic sclerotome). The grafted quail notochord, though visible morphologically, never stained for QCPN (Fig. 4I, J; ENC= ectopic quail notochord).

In experimental embryos with a notochord graft alone, Pax1 WMISH revealed ectopic sclerotome in the grafted region of all embryos (8/8 embryos; Fig. 4B, C, D, E), confirming the previous results. The ectopic sclerotome did not stain for QCPN, confirming its host origin (5/5 embryos, Fig. 4B-E). These results rule out accidental transfer of quail somite cells as a complicating factor, and confirm that a grafted notochord compresses the segmental pattern of the host sclerotome.

### 2.3 The periodicity of ectopic sclerotome is determined by the adjacent somites

**Fig. 5:**
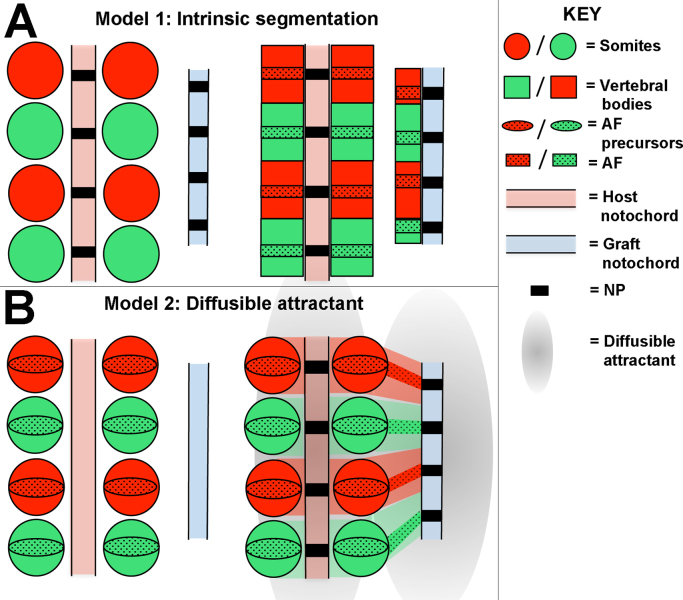
Two potential mechanisms by which ectopic sclerotome segmentation could be compressed. Both models show somites before (left) and after migration of the sclerotome to the notochord. **A. Model 1:***The notochord contains an intrinsic segmental pattern that instructs the position of the IVD within the sclerotome.* The quail notochord is released from tension when excised from the donor embryo, compressing this segmental pattern. This compressed segmental pattern is then translated into the vertebral bodies and IVDs after migration of the sclerotome to the midline. **B. Model 2:***The notochord contains no intrinsic segmental pattern, but secretes a diffusible attractant causing sclerotome within range to move towards the graft.* The pattern of AF and vertebral body precursors is intrinsically determined within the sclerotome. However this pattern is compressed after migration towards the graft due to either competition for space at the midline, or due to the smaller number of cells that have migrated towards the graft compared to the endogenous notochord.

The results of the notochord graft and ablation experiments suggest that the notochord is required for vertebral body segmentation and that it has the ability to alter the periodicity of adjacent sclerotome. Two possible mechanisms could explain this. First, *the notochord may possess a hidden segmental pattern that determines vertebral and IVD boundaries within the sclerotome* (Model 1) (Fig. 5A). The notochord is a rod of epithelial cells, under tension along its length. When a piece of notochord is excised from the quail donor, its length quickly shrinks as tension is released. If the notochord contains segmental information, this would also shrink to a more compact pattern, which could be responsible for the compressed pattern of ectopic sclerotome described in the previous section. Second, *the notochord may lack intrinsic segmental information, but secretes a diffusible factor that attracts sclerotome cells towards the ectopic notochord* (Model 2) (Fig. 5B). In this model, future AF cells within the sclerotome are specified autonomously within the somite. The action of the attractant could then compress this pattern in one of two ways. First, given that the range of the diffusible factor would be greater than the length of the notochord graft from which it is emitted, as sclerotome cells migrate towards the graft, they would be forced to occupy a smaller space, compressing the segments. Alternatively, the pattern may be compressed due to a smaller number of cells being recruited from the lateral somite towards the grafted notochord.

**Fig. 6:**
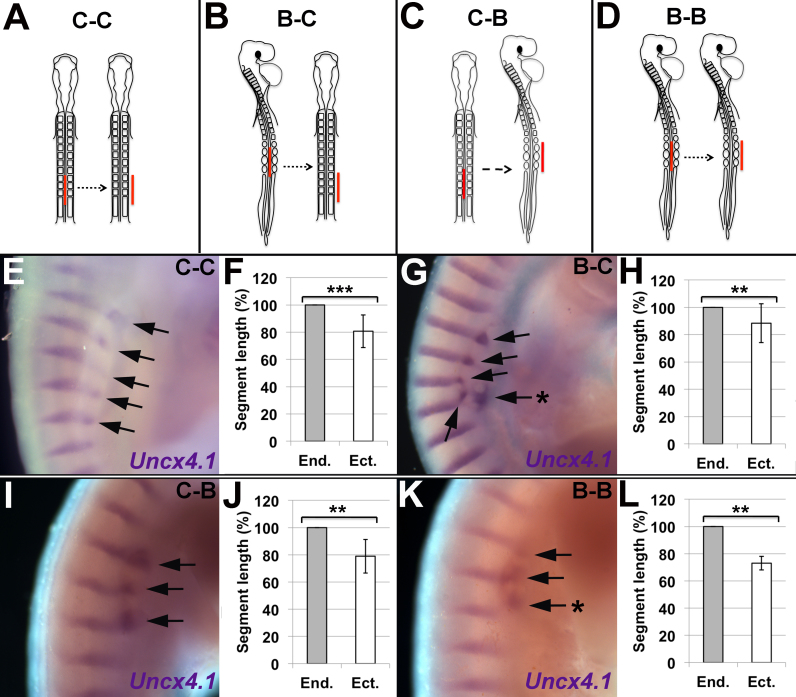
Inter-regional notochord grafts suggest that the periodicity of ectopic sclerotome is dependent upon somite size, not the axial region of the notochord. A-D. Schematics showing the four inter-regional notochord grafts carried out (red=grafted quail notochord). A. Cervical notochord grafted to cervical somites (C-C). B. Brachial notochord grafted to cervical somites (B-C). C. Cervical notochord grafted to brachial somites (C-B). D. Brachial notochord grafted to brachial somites (B-B). E-L**.** Images and quantification of HH stage 24–25 embryos, three days after notochord graft. E, G, I, K. WMISH for Uncx4.1 (purple) shows the segmental pattern of ectopic sclerotome resulting from each graft (black arrows = segments of Uncx4.1 expression; Black stars = segments of expression that are significantly out of phase with the endogenous expression pattern). Letters in top right corner refer to axial region of graft and host. F, H, J, L. Comparison of mean ectopic and endogenous segment length for each graft.

If Model 1 is correct and the periodicity of the sclerotome is influenced by intrinsic segmental information within the notochord, an identical length of notochord should give rise to the same number and periodicity of segments regardless of the periodicity of the adjacent somites. To test this, notochord grafts were carried out across regions with different somite size: mid-cervical somites are smaller than those in the brachial region. Notochord grafts were conducted between cervical and brachial regions, using embryos at HH 10–11 (10–13 somites) and HH 13–14 (19–22 somites) respectively (Fig. 6A–D). The pattern of ectopic sclerotome at HH 24–25 was compared across embryos stained for Uncx4.1 (Fig. 6E, G, I, K). The length of donor notochord was kept as constant as possible between all grafts.

At HH 24–25, 18 of the 21 grafted embryos showed ectopic sclerotome (Fig. 6E, G, I, K; black arrows). Quantification of segment length showed that in all cases, the ectopic sclerotome segments were compressed compared to the adjacent endogenous sclerotome (Fig. 6F, H, J, L), and this difference was found to be statistically significant (Table 1). Furthermore, a number of ectopic segments were seen to be out-of-phase with the endogenous segmentation pattern (Fig. 6G, K; black stars). This confirms the results of the previous notochord graft experiment.

**Table 1:** Summary of the mean percentage difference in segment length between the endogenous and ectopic sclerotome, three days after a notochord graft across cervical and brachial axial regions. Grafts are illustrated in Fig. 5.

Graft	Mean % difference in segment length	S.D.	Pairwise T-test
Cervical – Cervical (C-C)	19	12	t(13) = 6.21 p = 0.000032
Brachial – Cervical (B-C)	12	14	t(17) = 3.57 p = 0.002357
Cervical – Brachial (C-B)	21	12	t(8) = 5.01 p = 0.00104
Brachial – Brachial (B-B)	27	5	t(3) = 9.95 p = 0.00216

In embryos where a cervical or brachial notochord was grafted adjacent to the cervical somites, Uncx4.1 expression showed 3–6 segments of ectopic sclerotome (Cervical-Cervical [C-C]; Fig. 6E: 5/5 embryos; Brachial-Cervical [B-C], Fig. 6G: 5/6 embryos). In contrast, embryos where notochords from either region were grafted to the brachial somites consistently generated 3 segments of ectopic sclerotome (Cervical-Brachial [C-B]; Fig. 6I: 5/6 embryos; Brachial-Brachial [B-B]; Fig. 6K: 2/2 embryos). The segmental pattern of ectopic sclerotome did not change, irrespective of the region of origin of the notochord graft. Notochord grafts from the cervical region were 5–6 somitic segments long, whilst the same length of notochord from the brachial region spanned only 4 somites. However, both grafts consistently generated only three segments of ectopic sclerotome adjacent to the brachial somites. These results strongly suggest that the periodicity of the somites determines the segmental pattern of the ectopic sclerotome, dominating over any segmental pattern that may be present in the notochord. This is evidence against Model 1 (Fig. 5A). The results are consistent with Model 2, in which compression of a pre-determined segmental pattern within the sclerotome is achieved by sclerotome cell migration in response to a diffusible attractant from the notochord (Fig. 5B). The mid-cervical somites in the chick embryo are smaller than those in the brachial region. If the same length of notochord was grafted lateral to the somites in both of these regions, the radius of diffusion of the hypothetical attractant would remain constant between the two grafts, but the number of somites that sit within this radius (and are therefore able to respond) would be fewer in the brachial region. The brachial sclerotome that is attracted to the notochord graft therefore forms fewer, more widely-spaced segments than in the cervical region.

### 2.4 The sclerotome expands after 8 h exposure to a notochord graft

The above results suggest that the notochord secretes a diffusible attractant for sclerotome cells. Although we have shown that ectopic sclerotome derives from the chick host (not the quail graft), we do not know from where in the host these cells derive. Previous notochord graft studies over a shorter incubation time have shown that Sonic Hedgehog (Shh) and Noggin from the notochord induce the sclerotome. Normally, this induction occurs in the ventromedial somite, but a lateral notochord graft extends the sclerotomal domain into the lateral somite (Brand-Saberi et al., 1993, Pourquie et al., 1993, Fan and Tessier-Lavigne, 1994, Dietrich et al., 1997, McMahon et al., 1998). It seems likely therefore, that this lateral sclerotome is the source of our ectopic sclerotome seen after three days. However, it is possible that it is formed (in part) by induction of sclerotome in the lateral plate mesoderm (LPM) surrounding the notochord. The distinction between these two origins is important. If ectopic sclerotome is induced and recruited in the lateral somite, the resulting segmental pattern is a true compression of the original pattern established in the somites. If, however, it derives by induction in the LPM, the segmental pattern is established de novo. Only the former mechanism requires the action of an attractant secreted by the notochord.

**Fig. 7:**
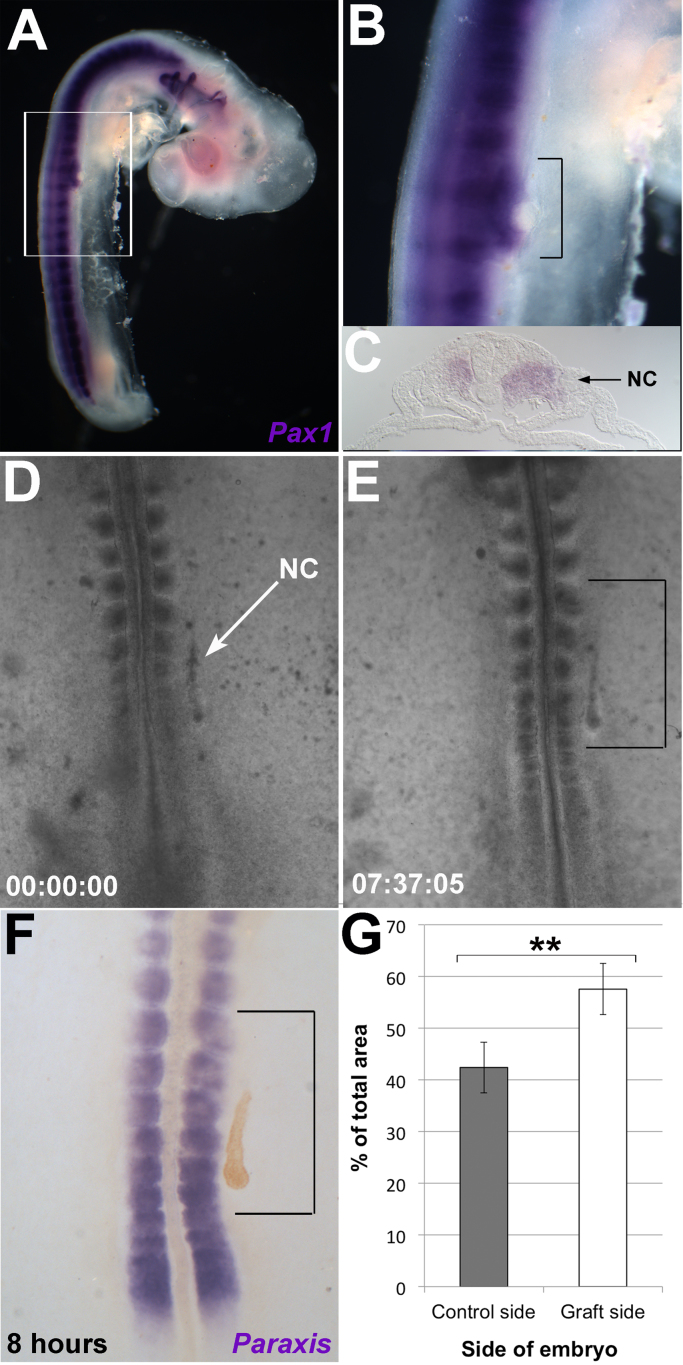
A notochord graft leads to expansion of the sclerotome prior to separation of endogenous and ectopic sclerotome. A. WMISH for Pax1 (purple) shows presence of ectopic sclerotome in a HH18 embryo, one day after a notochord graft. Ectopic sclerotome is continuous with the endogenous sclerotome. B. Higher magnification on boxed region of A. Black bracket indicates expanded *Pax1* expression. C. Transverse section of embryo one day after a notochord graft (NC), showing expansion of sclerotome into the lateral somite adjacent to the graft. D–E Time-lapse imaging of a developing embryo, in which a quail notochord (NC) has been grafted lateral to the somites and anterior PSM. D. Grafted embryo at 0 h incubation. E. Embryo approximately 7.5 h after the graft. Somites adjacent to the notochord graft (black bracket) expand towards the graft. **F.** WMISH for somite marker *Paraxis* (purple) on embryo shown in E, after 8 h incubation. The quail notochord graft (brown) was detected by an immuno-stain for the QCPN quail cell marker. *Paraxis* expression confirms that somites are expanded towards the graft. **G.** Quantification of somite area in response to a notochord graft. The mean total area of the four somites closest to a notochord graft, across six embryos, was compared to contralateral somites (control side), after eight hours exposure to a notochord graft. The total area of somites on each side of the embryo is expressed as a percentage of the total area of all eight somites measured per embryo. A paired sample T-test shows that the greater percentage area of somites on the graft side compared to the control side is statistically significiant (t(6) = 3.88, p = 0.008).

To distinguish between these two possibilities, we attempted to trace DiI and DiO-labelled somite cells adjacent to the grafted notochord. However, results were inconclusive because the dyes are washed out during the WMISH procedure for Pax1, required to identify ectopic sclerotome and because it is difficult to follow cell migration for 3 days. We therefore investigated the effect of a notochord graft on the sclerotome over a shorter time. The original notochord graft experiment (Fig. 2A) was repeated; after 24 h (HH 18), ectopic Pax1 expression was seen in the grafted region (Fig. 7A, B). This ectopic expression appeared as a ventrolateral expansion of the endogenous expression domain (Fig. 7B; black bracket), in contrast to the separate domain of expression typically seen two days later at HH24/25 (see Fig. 2B, C). In transverse section, ectopic sclerotome expression is seen in the lateral somite (Fig. 7C). Here, the epithelial sheet of Pax1-negative dermomyotome (seen in the contralateral somite), has been replaced by mesenchymal, Pax1-positive sclerotomal cells. No ectopic Pax1 expression was seen in the LPM surrounding the notochord graft. This strongly suggests that the ectopic sclerotome is recruited from the lateral somite.

All the experiments described above were carried out in ovo. However, embryos can be grown in New (1955) culture for about 24 h, which allows video time-lapse filming to observe the effect of a notochord graft. Still images are shown in Fig. 7D-E. Immediately after grafting (00:00:00), the implanted notochord can be seen lateral to the paraxial mesoderm on the right side of the embryo (Fig. 7D). After 7 h 37 min, a further four somites had formed. There was a clear change in the shape and size of the somites close to the notochord graft compared to the contralateral somites on the unoperated side (Fig. 7E; black bracket). This is most obviously seen in the two most rostral somites within the black bracket, which show a lateral expansion towards the notochord graft, most prominent in the caudal part of the somite. This expansion gives the impression that the somites are curved towards the graft. An overall lateral expansion could be seen in the more caudal bracketed somites (immediately adjacent to the notochord graft).

After filming, the embryo was analysed for Paraxis, a marker of the anterior PSM and somites (Burgess et al., 1995). QCPN immunostaining was then carried out to locate the quail notochord graft (Fig. 7F). Paraxis expression was also expanded laterally adjacent to the notochord graft compared to the contralateral somites, confirming the somite expansion observed in time-lapse movies. To quantify somite expansion, notochord grafts in culture were repeated and Paraxis staining used to mark the somites as in Fig. 7F. Embryos were analysed after 8 h’ incubation. The projected area of the four somites closest to the notochord graft (right) was compared to that of the contralateral somites that had not been exposed to a notochord graft (left). The area of somites adjacent to the graft was on average 15.2 ± 4.9% greater than that of the control somites after 8 h in culture (Fig. 7G; paired-sample T-test: t(6) = 3.88, p = 0.008), confirming a significant expansion of the somites. These experiments suggest that the notochord graft induces and recruits ectopic sclerotome cells from the lateral somite, confirming that segmentation of ectopic sclerotome at three days is a compression of the endogenous somite pattern. Furthermore, the observation that somites expand rostral to the graft (not just immediately adjacent to it), suggests that somite expansion is mediated by a diffusible molecule.

### 2.5 Notochord grafts eventually lead to the formation of ectopic cartilage

**Fig. 8:**
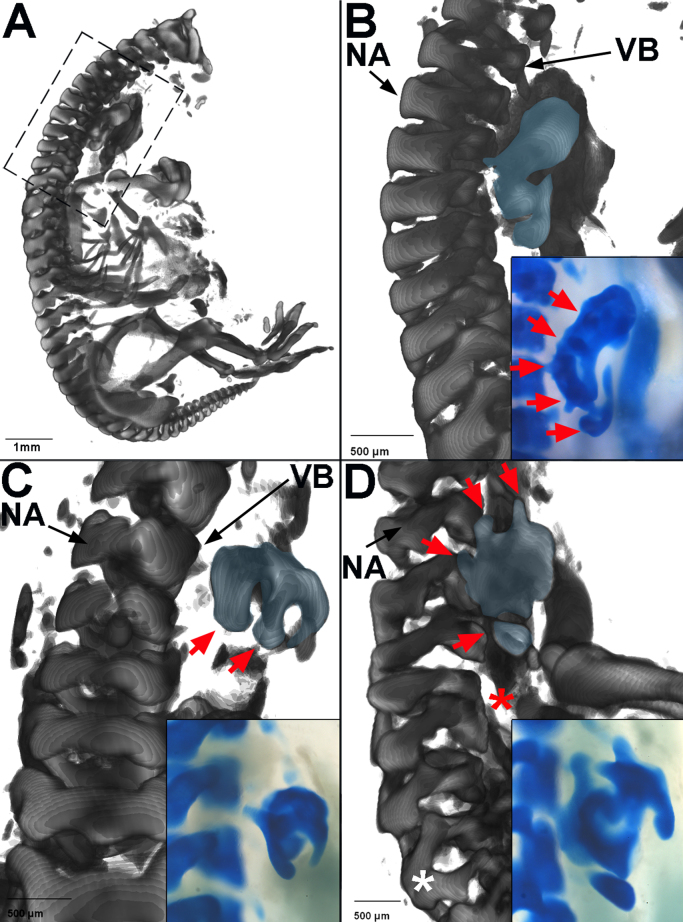
A notochord graft leads to formation of ectopic cartilage. A–D**.** OPT reconstructions of skeletal preparations of HH30–33 embryos, six days after a notochord graft. Ectopic cartilage is highlighted in blue. Inset images show bright field images of ectopic cartilage, stained with Alcian Blue. (NA=neural arch; VB=vertebral body, Red arrows= potential segmentation of cartilage) A. Lateral view of whole embryo (head removed) shows ectopic cartilage in grafted region. B. Zoom on boxed region of embryo shown in A. C. Second example of ectopic cartilage. Zoom on ectopic cartilage, which shows a ring-like morphology. D. Third example of embryo showing ectopic cartilage. Zoom on ectopic cartilage. This embryo shows disruption to the morphology of the endogenous vertebrae. (red star=fused vertebral bodies, white star = fused neural arches).

The above results demonstrate that the notochord can compress the segmental pattern of sclerotomal condensations, but does this affect the final pattern of vertebrae and IVDs? To address this, embryos with an implanted notochord (Fig. 2A) were incubated for a further 6 days (HH32–34). Skeletal preparations (Alcian blue staining) were analysed using OPT. Ectopic cartilage could be seen in the grafted region lateral to the endogenous host vertebral column on the right hand side (n = 5) (Fig. 8A-D; ectopic cartilage in OPT images is indicated by a semi-transparent blue overlay). The morphology of the ectopic cartilage varied dramatically between embryos. However, in all cases, the ectopic cartilage was not a continuous sheet but was arranged as discrete regions of strong Alcian blue staining, which may represent some degree of segmentation (Fig. 8B-D; Red arrows). However, it was not a perfectly normal pattern of vertebral elements. Various shapes and processes were visible and in one embryo, the ectopic cartilage formed ring-like structures that appeared to wrap around the grafted notochord (Fig. 8C; two rings indicated by red arrows). The endogenous vertebrae were unaffected, except in one embryo where fusions of some of the neural arches (Fig. 8D; white star) and vertebral bodies (Fig. 8D; red star) were seen posterior to the region of ectopic cartilage, perhaps caused by damage to the somites or neural tube during grafting.

### 2.6 A neural tube and notochord graft leads to formation of ectopic neural arches

**Fig. 9:**
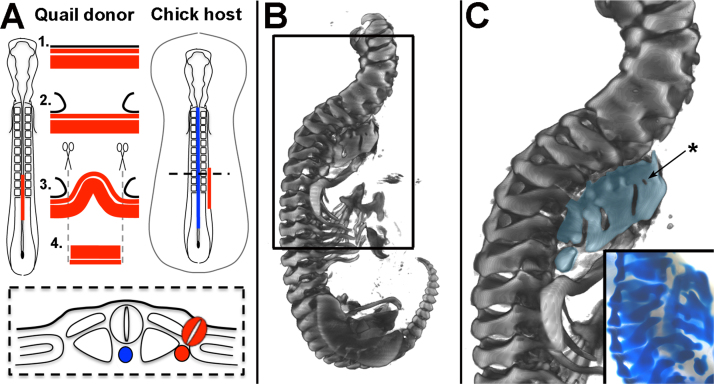
Ectopic cartilage resulting from a notochord and neural tube graft. A. Schematic diagram showing notochord and neural tube graft procedure (quail neural tube and notochord shown in red). B. OPT reconstruction of HH33 embryo skeletal preparation, six days after a notochord and neural tube graft. **C.** Zoom on boxed region of embryo in B. Ectopic cartilage shaded in blue, containing elements of neural arch-like morphology and a hole that resembles a foramen (star). Inset panel shows bright field image of ectopic cartilage, stained with Alcian Blue.

In the skeletal preparations of notochord grafted embryos, cartilage resembling vertebral bodies was seen but neural arch-like morphology was never observed. Together with the result that neural arches are largely unaffected by notochord ablation, this suggests that the notochord is not involved in patterning the neural arches. Studies in which the neural tube was ablated reported that in the absence of a neural tube, no neural arches form but vertebral bodies form normally (Watterson et al., 1954, Strudel, 1955, Teillet and Le Douarin, 1983). This result suggests that ventral and dorsal elements of the vertebral column are patterned independently and that the neural tube is required for neural arch segmentation. To test this further, the notochord and neural tube were grafted together, lateral to the host PSM (Fig. 9A). At HH33, ectopic cartilage was seen in the grafted region (Fig. 9B, C; Blue overlay=ectopic cartilage). The cartilage was more extensive than in embryos of the same stage with a notochord graft alone, and contained four or five elements with neural arch-like morphology (Fig. 9C; red arrows), with varying degrees of fusion between each element. The most anterior element contained a hole in the cartilage (Fig. 9C; star), resembling a foramen through which a segmental vein or artery might project. This suggests that signals from the neural tube induce and pattern the neural arches, consistent with neural tube ablation studies.

### 2.7 Does the notochord possess an intrinsic segmental pattern?

**Fig. 10:**
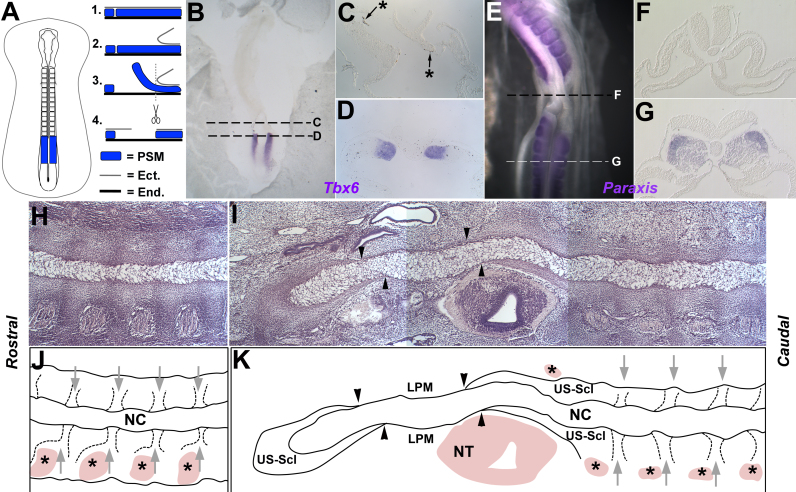
PSM ablations suggest the notochord cannot segment in the absence of the surrounding somites. A. Schematic diagram showing the PSM ablation procedure.(Left= dorsal view; right= sagittal view of steps 1–4 of ablation procedure). B–D. WMISH for Tbx6 (a marker of the PSM) in HH11–12 embryos fixed immediately after ablation, demonstrating successful ablation of the PSM from a portion of notochord. C. Transverse sections confirm the complete absence of Tbx6-positive cells in the ablated region (black star=remnants of ink used for contrast during ablation procedure). D. Transverse section shows that the PSM posterior to the ablated region was unaffected. **E–G.** WMISH for paraxis (marker of the somites) in embryos cultured overnight after PSM ablation, showing that PSM ablation led to a complete absence of somites in the ablated region. F. Transverse section confirming the absence of paraxis-positive cells in the ablated region, with lateral plate mesoderm moving medially to lie adjacent to the notochord. G. Transverse section showing that somites posterior to the ablated region form normally. H–I. Haematoxylin-stained coronal sections through notochord of a six day old embryo, four days after PSM ablation. The length of the ablated region is shown in two sections. H. Section rostral to ablated region. Notochord shows segmental pattern of swellings and constrictions that coincide with dark stripes in surrounding sclerotome which correspond to the future AF region. I. Section spanning ablated region (left) and further caudally (right). The area between black arrowheads shows absence of sclerotome and the notochord shows no signs of segmentation. Immediately anterior and posterior to this, the notochord is surrounded by apparently unsegmented sclerotome. Caudally, normal segmented sclerotome is seen surrounding the notochord and the segmental pattern of swellings and constrictions in the notochord resumes. J–K. Schematic diagram corresponding to sections H-I. NC = notochord, NT = neural tube, US-Scl = unsegmented sclerotome, LPM=lateral plate mesoderm, Black stars = dorsal root ganglia, grey arrows = stripes of high cell density in sclerotome corresponding to future AF.

The results of notochord grafts across regions support the idea that the chick notochord has no intrinsic segmental pattern. However, notochord ablation experiments here and elsewhere (Watterson et al., 1954, Strudel, 1955) suggest that the notochord is required for segmentation of the vertebral bodies and IVDs. This apparent contradiction might be reconciled by a model in which the initial segmental pattern of the somites is imprinted upon the notochord, after which the segmented notochord signals back to the sclerotome to position the annulus fibrosus and vertebral bodies. To test this, we asked whether the notochord can segment in the absence of adjacent somites. A portion of the PSM was ablated at HH 11–12 (Fig. 10A). To confirm successful ablation, WMISH for the PSM marker Tbx6 was performed on embryos fixed immediately after ablation. A gap in Tbx6 expression can be seen on either side of the midline, immediately posterior to the most caudal somite (Fig. 10B–D; n = 4). After overnight culture, the somite marker Paraxis showed complete absence of somites in the ablated region, whereas the more posterior somites formed normally (Fig. 10E–G; n = 3). In the ablated region, the LPM had moved into the gap to sit adjacent to the notochord (Fig. 10F). We were therefore confident that we could ablate the PSM successfully to eliminate segmented sclerotome in the operated region.

The first overt sign of notochord segmentation is the appearance of periodic swellings and constrictions along its length, which mark the position of the future IVDs (Lillie and Hamilton, 1952). To test whether the notochord can segment in the absence of PSM, we performed the PSM ablation described above and incubated embryos for a further 4 days (6 days total), when segmentation of the notochord can be visualised (Fig. 10A). Histology and haematoxylin staining (Fig. 10H, I; N = 1) showed that the sclerotome was absent in the operated region (Fig. 10I, K; region between black arrowheads). Instead, tissue resembling the lateral plate mesoderm is found adjacent to the notochord. Immediately rostral and caudal to this, a small amount of sclerotome tissue surrounds the notochord, but shows no sign of segmentation (Fig. 10I, K; US-Scl). Outside the operated region, the segmented sclerotome is seen surrounding the notochord (Fig. 10H-K), with regularly spaced stripes of higher cell density likely to correspond to the future annulus fibrosus (grey arrows). In all regions that still contain segmented sclerotome the notochord displays a regular pattern of swellings and constrictions (Fig. 10H-K). In contrast, where there is no sclerotome segmentation, the notochord lacks all signs of segmented swellings (Fig. 10I, K). This suggests that the pattern of periodic swellings of the notochord is dictated by the adjacent sclerotome.

## 3 Discussion

The vertebral column is derived from two embryonic tissues: the sclerotome (which gives rise to the vertebrae and annulus fibrosus (AF) of the IVDs) and the notochord (which forms the central nucleus pulposus (NP) of the IVDs). During development, the AF and NP form together to position an IVD at the boundary between successive vertebral bodies. To accomplish this, the sclerotome and notochord must adopt the same segmental periodicity. However, it is unclear what determines the position of the AF within each sclerotomal segment and likewise, what dictates the position of each NP along the length of the notochord.

We report that when the notochord is ablated, the segmental arrangement of vertebral bodies and IVDs does not form, consistent with previous ablation studies (Watterson et al., 1954, Strudel, 1955). When the PSM is ablated (removing the sclerotome from around the notochord), segmentation of the notochord is also lost. These experiments suggest that there is a mutual requirement of the sclerotome and notochord for the vertebral bodies and IVDs to segment in register, but which tissue dictates the position of each segment? The results of our notochord graft experiments suggest that segmental periodicity is ultimately determined by information intrinsic to the sclerotome, not the notochord. We find no evidence for an intrinsic segmental pattern within the chick notochord that dictates the position of the NP.

Together, our results are consistent with a model in which IVD position in chick (and hence the vertebral boundary) is determined by a series of reciprocal signalling events between the sclerotome and notochord. The initial segmental pattern is specified intrinsically within the sclerotome. This pattern is then imposed upon the notochord by the sclerotome. This segmentation initially manifests in the notochord as a series of swellings and constrictions until eventually notochordal cells are removed from the vertebral bodies and remain only in the NP at the centre of the IVD. After acquiring a segmental pattern, however, the notochord is required for the maintenance of segmentation within the surrounding sclerotome. If the notochord is absent, the IVD cannot form normally and adjacent vertebral primordia fuse. If the sclerotome is absent, vertebral and AF primordia cannot form and the notochord cannot segment.

### 3.1 Sclerotome cell attraction by the notochord

Notochord grafts led to the formation of ectopic sclerotome with a segmental periodicity that is compressed compared to the endogenous sclerotome. However, further analysis revealed that the periodicity of ectopic sclerotome is dictated by the periodicity of endogenous somites, arguing against the idea that the notochord has a covert segmental periodicity that dictates the position of vertebral boundaries (Model 1: Fig. 5A). How then, can we explain the compression of ectopic sclerotomes? We propose that it is the result of an attraction of adjacent sclerotome cells towards the notochord, which compresses the segments as it does so, probably aided by the immiscibility of R and C sclerotome cells (Stern and Keynes, 1987).

Given that the sclerotome migrates towards the midline to surround the notochord and neural tube, it seems likely that the midline structures emit a chemoattractant for the sclerotome. It is possible that relocation of the sclerotome is to some degree aided by mechanical forces: ventral closure occurs across this period of development and therefore significant changes to the geometry of the embryo accompany sclerotome migration. However, it has been shown that chemical inhibition of cytoskeletal contraction at this stage in development prevents migration of the sclerotome (Chernoff and Lash, 1981), reinforcing the idea that movement of sclerotome to the midline involves active cell migration. The notion that the notochord emits a chemoattractant is also supported by the observation that sclerotomal cells cultured on a collagen matrix actively migrate towards notochord explants in vitro (Newgreen et al., 1986).

Our experiments in New culture show that somites expand laterally in response to a notochord graft over a wide region. This also suggests the action of a diffusible molecule. One possible candidate is FGF4, which has been suggested to attract paraxial mesoderm to the midline during early neurulation (Yang et al., 2002). Another candidate is Sonic Hedgehog (SHH), which influences sclerotome cells in a number of ways including induction (Fan and Tessier-Lavigne, 1994), survival and proliferation (Fan et al., 1995, Teillet et al., 1998) of the sclerotome. Further work, perhaps using explant cultures, is required to elucidate the molecular basis of sclerotome attraction.

### 3.2 From sclerotome segmentation to segmented cartilage

Longer incubation of notochord grafted embryos showed that ectopic cartilage eventually forms mature cartilage elements. In some embryos, this cartilage resembled vertebral bodies and often displayed some degree of segmentation; however, it was far from a complete recapitulation of vertebral morphology. Therefore, although the notochord is required for formation of the vertebral bodies and IVDs, ectopic sclerotome was not able to translate its segmental pattern into a normal arrangement of vertebral bodies and IVDs. In the ectopic location, the structures that surround the notochord and sclerotome vary dramatically compared to the normal midline. Furthermore, whereas the vertebrae and IVDs normally form from bilateral sclerotomes, ectopic cartilage forms from sclerotome on only one side. These differences may all contribute to a mechanical and signalling environment that affects the shape of the vertebral elements.

Interestingly, when the neural tube and notochord are grafted together, a more complete recapitulation of vertebral morphology is seen. The presence of neural arches in the ectopic structures suggests a role for the neural tube in neural arch patterning. It also demonstrates that vertebral bodies/IVDs and neural arches develop independently, despite having a common origin in the sclerotome. These findings agree with previous reports that neural arches are absent after neural tube ablation, whereas notochord ablation interferes with segmentation of the centra (Watterson et al., 1954, Strudel, 1955, Teillet and Le Douarin, 1983). Furthermore, the independence in their development is reflected in the fossil record, which suggests that vertebral bodies and neural arches have been gained and lost independently throughout vertebral evolution (Fleming et al., 2015).

### 3.3 The role of the sclerotome in NP formation

Our PSM ablation experiment suggests that the sclerotome imprints its segmental pattern onto the notochord, specifying the NP to form in register with the AF. Several pieces of evidence suggest that this is achieved (at least in part) by mechanical cues, where the sclerotome in vertebral body regions physically ‘squeezes’ the notochordal cells into the future NP (Aszodi et al., 1998, Smits and Lefebvre, 2003, Choi and Harfe, 2011). This is consistent with the results of our PSM ablation: the notochord does not adopt a segmental pattern of swellings because there is no sclerotome to create the physical force that segments the notochord. It may also explain why no gene has been found to be expressed in a segmental pattern in the amniote notochord. If notochordal segmentation is the result of cell-rearrangements dictated by the sclerotome, no underlying molecular segmentation within the notochord would be required.

It is important to note that ablation of the PSM removes not only the sclerotome, but also all somite derivatives in this region, which include muscle, blood vessel, connective tissue and dermal precursors (Christ and Ordahl, 1995, Christ and Scaal, 2008). Given that all of these have a segmental organisation arising from the somites, it is possible that it is their absence, rather than the sclerotome, that results in loss of notochord segmentation. To answer this question fully, an analysis of notochord segmentation in embryos lacking R-C somite polarity would be required (for example in Mesp2/Ripply1/2 mouse mutants (Takahashi et al., 2013)). However, given that no evidence was obtained for an intrinsic segmental pattern within the chick notochord, our interpretation that notochord segmentation is imparted by the sclerotome seems the most likely scenario.

### 3.4 Specification and maturation of the annulus fibrosus

One question that remains unanswered is exactly when and where sclerotomal cells become committed to either a vertebral body or AF fate. Our results suggest that some kind of segmental pattern is determined intrinsically within the sclerotome, and that this pattern is imposed upon the notochord, but whether this pattern represents a commitment to these opposing cell fates is unclear. After notochord ablation, we saw no regions of weak or absent Alcian blue staining in the ablated region (which typically indicates the position of the IVDs), suggesting that the notochord is required for the development of the AF. There are two likely explanations. The first is that the notochord, having acquired a segmental pattern from the surrounding sclerotome, signals back to the sclerotome to specify cells to an AF fate. The other possibility is that AF cells are specified within the sclerotome independent of the notochord, but that signals from the notochord are then required to promote the proliferation and/or survival of these AF cells.

Studies in which sub-regions of the sclerotome were traced into the axial skeleton propose different origins of the AF, including the caudal sclerotome (Takahashi et al., 2013), rostral sclerotome (Goldstein and Kalcheim, 1992) and the core cells that reside in the somite lumen (somitocoele) (Huang et al., 1996). Interestingly, it has been reported that when somitocoele cells are grafted to the sclerotome that will form the vertebral bodies, they do not form AF but instead take on a fate appropriate to their new location (Senthinathan et al., 2012). This supports the idea that the population of sclerotomal cells that form the AF are not committed to such a fate until they have received signals from the notochord. It is not clear whether AF cells are unique in their capacity to respond to signals from the notochord or whether they only do so by virtue of their position adjacent to the NP after the notochord has segmented. If the latter, no pre-specification of AF and vertebral body sub-domains would be required.

Pax1, which is initially expressed by all sclerotome cells, becomes restricted to the future AF region of the sclerotome at stage HH 29 in the chick (Deutsch et al., 1988, Wallin et al., 1994, Senthinathan et al., 2012). Although this segmental expression marks the region where the future AF will form, it is unclear whether Pax1-expressing cells are committed to an AF fate. Another study involving notochord ablations in chick found that this later segmental expression of Pax1 persists in the absence of the notochord (Senthinathan et al., 2012). This at first appears to contradict findings here and elsewhere (Watterson et al., 1954, Strudel, 1955), that notochord ablation abolishes segmentation at the later stage of vertebral body and IVD formation. Our model reconciles these results: the notochord is not required for specification of an initial segmental pattern within the sclerotome, but plays a later role in maintaining this pattern as the mature vertebral bodies and AF develop. It is possible that in the absence of these signals, cells that would normally form the AF adopt the same fate as the chondrocytes of the vertebral bodies by default and adjacent vertebral bodies fuse.

Evidence for this comes from a mouse knockout of Sox5/6, which is expressed in both the notochord and sclerotome during vertebral development. Interestingly, a segmental pattern develops normally in the sclerotome in Sox5/6 knockout mice (as shown by the restriction of Pax1 to this region), but Pax1-expressing cells ultimately form cartilage rather than AF (Smits and Lefebvre, 2003). This supports the idea that signals from the notochord are required to determine the fate of AF cells. However, it is still possible that Pax1-expressing cells are committed to an AF fate at an earlier stage, but that in the absence of survival or proliferative signals from the notochord (regulated by Sox5/6), these cells do not survive to form mature AF. Again, Sonic Hedgehog (SHH) is a likely candidate for such a signalling molecule, as it is expressed by the notochord and is known to promote proliferation of sclerotomal cells earlier in development (Fan et al., 1995). Indeed, Shh in the mouse notochord has been implicated in the regulation of mouse IVD formation (Choi and Harfe, 2011, Choi et al., 2012). However, given that it is expressed uniformly along the notochord, it is difficult to see how it could influence cell fate or proliferation/survival of the sclerotome in a segmental manner. One possibility is that the signal is uniform, but only those cells specified to become AF can respond.

### 3.5 Notochord segmentation in fish

In contrast to our results in chick, the sclerotome appears to be dispensable for vertebral body segmentation in some teleost fish. Evidence for this comes from the zebrafish *fused somites* mutant, where loss-of-function of Tbx24 leads to loss of R-C polarity (van Eeden et al., 1996, Nikaido et al., 2002) and fusion of somites. However, despite the lack of somite segmentation, vertebral bodies still form in a regularly-spaced, segmented arrangement, indistinguishable from wild type fish (van Eeden et al., 1996, Fleming et al., 2004). Here, something other than the sclerotome must be controlling segmentation.

The anatomy and development of the teleost spine is somewhat different to the situation in amniotes. The teleost sclerotome is much smaller, with most of the somite cells going on to form myotome (Morin-Kensicki and Eisen, 1997). The notochord also differs in the two vertebrate groups. In most amniotes, the notochord is comprised only of large, vacuolated cells and an acellular ECM sheath. In teleosts, the large vacuolated notochordal cells are surrounded by a layer of small, non-vacuolated, epithelial cells known as *chordoblasts*. These differences are reflected in the anatomy and development of the vertebral column. In teleosts, the vertebral bodies consist of the inner *chordacentra*, which initially form within the ECM sheath surrounding the notochord, and the outer *perichordal centra*, which directly ossify peripheral to the notochord around the chordacentra (Fleming et al., 2015). Experiments in zebrafish (Fleming et al., 2001, Fleming et al., 2004) and Atlantic salmon (Grotmol et al., 2003, Wang et al., 2013) have suggested that the inner chordacentra are not derived from the sclerotome, but instead might involve secretion of a mineralised matrix from the outer layer of notochordal chordoblast cells. The sclerotome gives rise to the perichordal centra, which follow the same segmental pattern as the underlying chordacentra (Grotmol et al., 2003, Fleming et al., 2004).

Other studies in zebrafish and salmon have suggested that not only does the notochord contribute cells to the vertebral bodies, but it also dictates the position of the vertebral boundaries. Laser ablation of the notochord in “segmentally reiterated” positions along the length of the notochord leads to loss of vertebral centra in these regions (Fleming et al., 2004). Furthermore, the first sign of segmentation in the axial skeleton of the Atlantic salmon is a change in the polarity of chordoblast cells of the notochord in a segmental pattern along the axis (Grotmol et al., 2003), which express Alkaline Phosphatase and later secrete the bony matrix of the chordacentra (Grotmol et al., 2005). Comparison of the notochordal transcriptome at time points spanning chordacentrum formation in the Atlantic salmon (*Salmo salar)* has revealed potential mechanisms for the regulation of segmental patterning, including the inhibition of mineralisation in the region of the intervertebral ligament (the fish equivalent of the IVD) by Collagen 11a2, potentially regulated by the canonical Wnt pathway (Wang et al., 2014). More recently, a study in zebrafish suggested a similar mechanism in which alternating domains of Collagen 9a2 and Entpd5a form sequentially from head to tail along the notochordal sheath. The Entpd5a domains mineralise (forming chordacentra) then specifically recruit sclerotomal osteoblasts to form the outer perichordal centra. The Col9a2 positive population marks the position of the intervertebral ligaments and does not recruit osteoblasts. RNA sequencing of the two sheath populations revealed that the Notch signalling pathway regulates the formation of mineralised segments (Wopat et al., 2018). Together, this is compelling evidence that the notochord possesses a segmental pattern in these teleost species, which lays the foundation of centrum periodicity.

The profound differences in how vertebral body segmentation is achieved across the vertebrates raises an important question – was the notochord segmented in the last common ancestor of the vertebrates? Unfortunately, our understanding of vertebral segmentation is limited to just a few “model” organisms, making it difficult to come to a consensus regarding the primitive state. The segmental pattern seems to be restricted to the outer layer of notochord-associated chordoblast cells in teleost fish, which most amniotes lack. There is no evidence that the inner vacuolated cells are able either to generate or to pattern the vertebrae. It would be reasonable to speculate, therefore, that the loss of chordoblasts in amniotes resulted in a loss of notochord segmentation, compensated for by the emergence of a much larger sclerotome with a greater role in dictating the segmental pattern. Interestingly, in the reptile Tuatara, epithelial cells have been described surrounding the notochord, raising the possibility that chordoblasts are not completely absent in all amniotes (Howes and Swinnerton, 1901). Furthermore, fate mapping in the skate (*Leucoraja erinacea*) found that the vertebrae are derived entirely from the somites in this species, despite the presence of an epithelial layer of cells surrounding the notochord, presumably equivalent to the teleost chordoblasts (Criswell et al., 2017). Also, analysis of vertebral body formation in the teleost Medaka found that sclerotomally-derived osteoblasts invade the notochordal sheath prior to formation of the chordacentra, suggesting (but not conclusively proving) that chordacentra are derived from the sclerotome in this species (Renn et al., 2013). It may be true that chordoblasts are required for the notochord to generate and pattern vertebral bodies; however, these results suggest that chordoblasts can be present without them performing this function.

### 3.6 Conclusions

Through a series of micromanipulations of the notochord and PSM in the chick embryo, we have shown that the segmental periodicity of the vertebral bodies and IVDs is determined by the sclerotome and find no evidence for an intrinsically-segmented notochord in amniotes. However, our results suggest that the sclerotome imposes its segmental pattern upon the notochord, ensuring that the notochordal and sclerotomal portions of the IVD segment in register. Although not involved in establishing a segmental pattern, we find that the notochord is required to reinforce the pattern established by the sclerotome to generate the alternating pattern of vertebral bodies and IVDs along the spine. Together, this suggests that sequential pattern-forming events are involved in generating the final pattern of the segmented vertebral column.

## 4 Materials and methods

### 4.1 Embryology

Fertile eggs from domestic fowl (*Gallus gallus*, Brown Bovan Gold; Henry Stewart&Co., UK) and Japanese quails (*Corturnix japonica*; B. C. Potter, Rosedean Farm, UK or Blue Bridge Engineering Limited, Essex, UK) were incubated at 38 °C in a humidified incubator and staged (Hamburger and Hamilton, 1951) (HH). Ca^2+^/Mg^2+^-free Tyrode's saline solution was used in all operations. Most operations were performed in ovo as described (Stern, 1993b), with the exception of notochord graft operations in Fig. 7D–F, which were performed in New culture as described (New, 1955, Stern, 1993b). After preparation for operation in ovo or in New culture, manipulations were performed under a dissecting microscope and an external fibre optic light source was positioned with the direction of light running parallel to the plane of the embryo, enabling visualisation of the relief of the embryo. Operations were facilitated using Trypsin (0.12% w/v) solution in Tyrode's as described (Stern, 1993b). After incubation to the desired stage, embryos were removed from the egg or membrane and washed briefly in Ca^2+^/Mg^2+^-free phosphate-buffered saline (PBS). For skeletal preparations, embryos were fixed in 95% ethanol for three days at 4 °C. For all other procedures, embryos were fixed in 4% paraformaldehyde (PFA) overnight at 4 °C.

#### 4.1.1 Notochord ablation

The notochord ablation procedure is illustrated in Fig. 1A. Two parallel cuts were made in the ectoderm on either side of the neural tube at HH11–12 using a hypodermic syringe needle (25 Gauge). The Tyrode's saline was then removed and replaced with a standing drop of Trypsin solution. The midline of the embryo could then be accessed, with the neural tube uppermost (Fig. 1A, step 1). The neural tube was moved gently from side to side using the back of the needle until the ventral neural tube could be lifted away to access the notochord beneath (Fig. 1A, step 2). The notochord was then moved from side to side in the same way as the neural tube and lifted free from the underlying endoderm (Fig. 1A, step 3). Here, it was essential that the notochord was moved gently, allowing the trypsin to enter below the notochord until it was freed without breaking the endoderm beneath. The free portion of notochord was then excised using the sharp point of the syringe needle, and the neural tube replaced to its original position (Fig. 1A, step 4). Sham notochord ablation experiments consisted of freeing and replacing the notochord.

#### 4.1.2 Notochord grafts in ovo

The notochord graft procedure is illustrated in Fig. 2A. Donor quail embryos were collected in Tyrode's and pinned out on a silicone-coated Petri dish (Sylgard), ventral surface uppermost (Fig. 2A, step 1). The embryo was then submerged in Trypsin and the endoderm gently peeled from the notochord at the level of the rostral PSM and caudal somites using a hypodermic needle (Fig. 2A, step 2). The notochord beneath was then moved gently from side to side (using the same action as described for notochord ablations) until it lifted free from the neural tube beneath (Fig. 2A, step 3). Again, it was important to do this gently, allowing Trypsin to enter and free the notochord cleanly. A portion of notochord was then excised using the needle (Fig. 2A, step 4) and transferred to a Petri dish in 3:1 Tyrode's : albumen mixture using a P20 Gilson pipette. The notochord was submerged in Tyrode's until ready for grafting. Next, the chick host was prepared for in ovo operation. A small incision was made through the ectoderm and into the space between the PSM and lateral plate mesoderm (LPM), at an R-C level around three somites in length caudal to the rostral edge of the PSM. A small “tunnel” was then made for the notochord graft by inserting the tip of a sharp steel insect pin (size A1) into the incision and pushing it rostrally through the PSM/LPM border as far as the third caudal somite. The insect pin was then carefully removed the same way. The quail notochord graft was then transferred to the host embryo in the Tyrode's/albumin mixture using a P20 Gilson pipette and carefully fed into the tunnel using the syringe needle (Fig. 2A, step 5). Sham operations were carried out by preparing chick hosts as above but with no notochord grafted.

The stage of embryos used for grafts varied according to the axial region to be studied. For grafts from and to the cervical region, quail and chick embryos at HH 10–12 were used (10–16 somites). For the brachial region, embryos at HH 13–14 (19–22 somites) were used. The notochord was always removed from a region spanning the somite/PSM boundary and grafted to the equivalent level in the chick host, to match the developmental ‘age’ of the notochord and somites regardless of the region. The length of notochord excised spanned 4–6 somites.

#### 4.1.3 Notochord and somite grafts

Notochord and somite grafts were carried out as described above for notochord grafts, except that a single quail somite was also grafted into the LPM, lateral to the notochord graft.

#### 4.1.4 Notochord grafts in New culture

For notochord grafts in New culture, donor quail notochords were collected as described above (Fig. 2A, step 1–5). Host chick embryos were then prepared for New culture and submerged in Tyrode's. Similar to the previous description, a small tunnel was made for the notochord graft at the PSM/LPM border. However, as embryos in New culture are positioned with their ventral surface uppermost, the initial incision was made through the endoderm, not the ectoderm as in ovo. After grafting of the notochord and removal of excess Tyrode's, embryos were cultured at 38 °C in a humidified chamber. For time-lapse imaging, the Petri dish containing the embryo in culture was sealed using Parafilm and incubated at 38 °C. Time-lapse imaging was carried out using an Olympus inverted microscope and Simple PCI software. Images were taken at 10 min intervals.

#### 4.1.5 Notochord and neural tube grafts

The notochord and neural tube graft procedure is illustrated in Fig. 9A. As for notochord grafts, donor quail embryos at HH10–11 were collected in Tyrode's and pinned out on a silicone (Sylgard)-coated Petri dish, ventral surface uppermost (Fig. 9A, step 1). The embryo was then submerged in Trypsin and the endoderm gently peeled from the notochord at the level of the rostral PSM and caudal somites using a hypodermic needle (Fig. 9A, step 2). The notochord and neural tube beneath were then moved gently from side to side, taking care that they didn’t separate, until they lifted free from the ectoderm beneath (Fig. 9A, step 3). The notochord and neural tube were then excised as a unit using the needle (Fig. 9A, step 4) and transferred to a Petri dish in a 3:1 Tyrode's: albumen solution using a P20 Gilson pipette. The notochord and neural tube were submerged in Tyrode's until ready for grafting. The chick host at HH10–11 was prepared and grafted as described for notochord grafts in ovo, except that a wider tunnel was made using the insect pin to accommodate the extra neural tube. The neural tube and attached notochord were then grafted, ensuring that the tissues remained attached to each other. Grafted embryos were incubated to HH33 and analysed by skeletal preparation as described below.

#### 4.1.6 PSM ablations

The PSM ablation procedure is illustrated in Fig. 10A. Chick embryos were incubated to HH 11–12. After preparation for in ovo surgery, Tyrode's was removed and replaced with a standing drop of Trypsin solution (Fig. 10A, step1). Two cuts were made in the ectoderm using a hypodermic needle following the medial edge of the PSM on either side of the neural tube. The ectoderm was then peeled free from the PSM carefully (Fig. 10A, step 2) and two additional parallel cuts made in the LPM following the lateral edge of the PSM. Working from rostral to caudal, the PSM was freed from the underlying endoderm by carefully lifting it up and down and from side to side using a hypodermic needle (Fig. 10A, step 3). Here, is was important to move the PSM very gently, allowing Trypsin to enter the space below and free it without breaking the underlying endoderm. After freeing a portion of PSM around 4–6 somites in length, a final transverse cut was made laterally across the PSM to excise the tissue (Fig. 10A, step 4). This was repeated for the contralateral PSM.

### 4.2 Whole-mount in-situ hybridisation (WMISH) and staining

WMISH was carried out essentially as described (Stern, 1998), with the following modifications to account for the later stage of the embryos. After fixation, embryos were bleached in 6% H_2_O_2_ in methanol for one hour at room temperature. Proteinase K digestion was optimised according to the stage of the embryos. As a general rule, embryos were digested for about 1 min per HH stage up to HH17. Thereafter, HH 18 embryos were digested for 25 min and HH stage 24/25 embryos digested for 40 min. Embryos in New culture were more fragile and therefore digested for only 6 min, regardless of stage. After antibody incubation, 5 × 1 h TBST washes were followed by a final overnight wash at 4 °C. The probes used were Pax1, Scleraxis, Paraxis and Tbx6. For Uncx4.1, a 650 bp fragment was amplified from cDNA derived from the trunk of a HH 25 chick embryo, using primer pairs F: GGTGGGGTAGAGCAAGAAGT and R: CGGACGTGTTTATGCGAGAG and GoTaq G2 DNA polymerase (Promega) and verified by sequencing before cloning into pGEM-T Easy.

Immunohistochemistry was carried out after WMISH, essentially as described (Stern, 1993a, Stern, 1998), using HRP-conjugated secondary antibodies and peroxidase detection using 3,3-diaminobenzidine (DAB) as substrate. Embryos were incubated in QCPN (anti-quail) antibody (1:5) for 3–5 days at 4 °C and in goat anti-mouse HRP-conjugated secondary antibody (Jackson) (1:1000) for 2–3 days.

For skeletal preparations, embryos were fixed in 95% ethanol and stained using Alcian Blue and Alizarin Red S (Sigma) using the method described for E17/18 mouse embryos (McLeod, 1980). OPT imaging was used to obtain 3-d images of skeletal preparations, using a Bioptonics OPT scanner 3001 M. Embryos were prepared as described (Sharpe et al., 2002).

Histological sections of Paraplast embedded embryos were cut at 10 µm and stained using Harris's Haematoxylin (Sigma).

### 4.3 Quantification of segment length

The rostro-caudal length of normal and ectopic segments was measured from images of embryos stained for Uncx4.1 or Scleraxis, 3 days after a notochord graft. The length of each segment was measured as the space between the caudal boundaries of adjacent Uncx4.1- or Scleraxis-expressing domains. In normal somites, expression of these markers spans the entire dorsoventral extent of each sclerotome. For consistency, all measurements were taken at the level of the notochord. Embryos were photographed in whole mount. The distance between consecutive ectopic segments was measured, along with that of the endogenous segments immediately adjacent, using Fiji (Schindelin et al., 2012). Ectopic segment lengths were expressed as a percentage of the corresponding normal segment lengths. Significance was estimated using a paired Student's T-test.

### 4.4 Quantification of somite area

To measure somite size after exposure to a grafted quail notochord, embryos were stained for Paraxis by WMISH to mark the somites and then immunostained for the quail nuclear marker QCPN to detect the graft. Embryos were photographed in whole-mount, maintaining the same magnification across all images. The four somites closest to the notochord graft were chosen for measurement. The projected total area of these four somites was calculated from the 2D bright field images using Fiji (Schindelin et al., 2012). The four somites on the contralateral side of the midline that had not been exposed to a notochord graft were also measured as a negative control. The total area of the eight measured somites (four bilateral pairs) was calculated, and the data normalised for size variation between embryos by converting the somite area of each side to a percentage of the total somite area for both the graft and contralateral side.
